# Immune-inflammatory responses in the elderly: an update

**DOI:** 10.1186/s12979-018-0117-8

**Published:** 2018-03-02

**Authors:** Giulia Accardi, Calogero Caruso

**Affiliations:** 1 Department of Pathobiology and Medical Biotechnologies, University of Palermo, Palermo, Italy; 2 Sezione di Patologia generale, Dipartimento di Biopatologia e Biotecnologie Mediche, Corso Tukory 211, 90134 Palermo, Italy

**Keywords:** Allergy, Cancer, Elderly, Immunosenescence, Immunestimulation, Inflammation, Vaccination

## Background

In Western countries the number of persons 65 years or older is most rapidly increasing. The percentage of people over 60 in the world has risen from 9.2% in 1990 to 11.7% in 2013 and, according to several estimates, it will reach 21% by 2050. It is expected to exceed the number of children for the first time in 2047. In addition, the number of persons above 80 years is projected to increase even more dramatically from a worldwide total of 125 million in 2015 to 434 million in 2050. Therefore, the problem of the elderly health is now a priority in medicine [1].

In May 2012, a group of scientists and clinicians developed a consensus statement to highlight the importance of a common view on ageing and healthy lifespan [2]. As reported in the panel, ageing is most likely one component of life, which first emerged in economically developed countries and results from a breakdown of self-organizing system and reduced ability to adapt to the environment. Ageing processes are defined as those that amplify the vulnerability of subjects, as they become older, to the factors that finally lead to death. In Western countries, the mortality rate increases 25 times more rapidly in individuals over 60 years old compared to people aged 25–44. Causes of death in aged are increased compared to individuals between 25 and 44 years old: cancer 43-fold, pneumonia and influenza 89-fold, heart diseases 92-fold, and stroke and chronic lung disease greater than 100-fold. These data suggest a key role for immunity in the survival of the elderly because resistance to these diseases depends at least in part on optimal immune function [3].

As reported in the Encyclopaedia of Immunobiology [4], in aged people, several changes of both innate and acquired immunity have been described and viewed as deleterious, hence the term immunosenescence. Thus, immunosenescence reflects age-related declines in immune function at the cellular and serological level. However, immunosenescence is a complex process involving multiple reorganizational and developmentally regulated changes, rather than simple unidirectional decline of complete immune function. On the other hand, some immunological parameters are commonly notably reduced in aged people. The principal immunosenescence hallmarks are represented by thymus involution with decreased new T cell generation and hematopoietic stem cell dysfunctions, decreased naïve and increased memory lymphocytes with accumulation of dysfunctional senescent cells with shortened telomeres. Defects in apoptotic cell death, mitochondrial function and stress responses, and malfunctioning of immune regulatory cells are also observed [5–7].

Immunosenescence is linked not only to the functional decline associated with the passage of time but also to antigen burden to which an individual has been exposed during lifetime. Therefore, a senescent immune system is characterized by continuous reshaping and shrinkage of the immune repertoire by persistent antigenic challenges. These changes lead to a poor response to newly encountered microbial antigens, including vaccines, as well as to a shift of the immune system towards an inflammatory, autoimmune, Th2 profile. This immune dysregulation provides the background for an increased susceptibility to autoimmune diseases, cancer, metabolic diseases, osteoporosis, neurological disorders, as well as to allergic inflammation and infections. Accordingly, the severity of many infections is higher in the elderly compared to younger adults and infectious diseases are frequently associated with long-term complications and frailty [4–8]. Reciprocally, an immune good function is tightly correlated to health status, as depicted in centenarian offspring [9, 10].

The chronic antigenic stress, which affects the immune system throughout life with a progressive activation of macrophages and related cells, contributes to determine an inflammatory status. The process of maintaining life for the individual is a constant struggle to preserve integrity. This can come at a price when immunity is involved, namely systemic inflammation. Inflammation is not per se a negative phenomenon. In fact, in response to cell injury elicited by trauma or infection the inflammatory response sets in, constituting a complex network of molecular and cellular interactions directed to facilitate a return to physiological homeostasis and tissue repair. If tissue health is not restored or the inflammatory trigger is not cleared, acute inflammation may become a chronic condition that continuously damages the surrounding tissues. The collateral damage caused by this type of inflammation usually accumulates slowly, sometimes asymptomatically for years but can eventually lead to severe tissue deterioration. Our immune system is, in fact, quite efficient in fighting acute infections in young people, but not particularly efficient in responding to chronic stimuli, especially when they occur late in life. This chronic state of low-grade inflammation, called inflamm-ageing, is characterized by increased levels of pro-inflammatory cytokines and acute phase proteins, which increase is a worse prognostic factor for all causes of death. This inflamm-ageing state is implicated in the pathogenesis of several age-related inflammatory diseases, such as atherosclerosis, diabetes, cancer and Alzheimer’s disease [11, 12].

Inflamm-ageing status does not depend only on immunosenescence, but other factors play a role. One cause of inflamm-ageing is represented by damaged macromolecules and endogenous host-derived cell debris that accumulates over time and, as a consequence of their increased production and/or inadequate elimination, are sources of chronic tissue damage [13]. Cellular senescence consists in the irreversible cell cycle arrest generally due to telomeres shortening or cellular DNA damage and is central in the inflamm-ageing process. Moreover, senescent cells secrete various extracellular factors, including inflammatory cytokines and chemokines that can enhance and propagate senescence with autocrine and paracrine modality. Therefore, senescent cells contribute to the pro-inflammatory status of ageing [14]. Gut microbiota is a further player in the induction and maintenance of immunoregulatory circuits and tolerance. The alteration of gut microbiota composition, named dysbiosis, can determine immune dysregulation, and subsequent low-grade chronic inflammation also due to the systemic effect of bacteria lipopolysaccharides [15].

The increasing incidence of obesity represents a great challenge to global health. Along with cardiovascular disease and diabetes several studies also show an increased cancer risk in this population. Indeed, obesity is associated with an alteration of adipocytes functional activity that leads to a chronic and systemic inflammatory state. This inflammation is currently considered one of the main molecular links that foster cancer development in obese patients [16, 17].

However, the most relevant role is played by the dietary pattern. The Western-type diet, high in red meat, high-fat dairy products, refined grains and sugars, has been associated with higher levels of C-reactive protein (CRP) and interleukin(IL)-6. On the other hand, Mediterranean diet and more in general diets high in fruit and vegetable intake have been associated with lower levels of inflammation. Several researches have also associated specific nutrients with different level of inflammatory markers. The impact of different nutrients on the systemic inflammation has been experimentally condensed into one-dimensional numeric values. The “dietary inflammatory index” (DII) weights each major macronutrient and multiple micronutrients on the basis of their general pro-inflammatory effects, as measured, for example, by assessment of CRP in serum [17, 18].

Ageing is the major risk factor for cancer development. In the relationship between cancer and ageing, several factors play a role. Inflammation is the most relevant [19]. As previously stated, one hallmark of the ageing process is, in fact, represented by inflamm-ageing [20] and inflammation is also one hallmark of cancer [21]. In fact, chronic inflammatory diseases increase the risk of some cancers and strong epidemiological evidence exists that anti-inflammatory drugs are potent chemopreventive agents. The microenvironment of cancer contains many different inflammatory cells and mediators; the targeting of these factors in models decreases the development, growth and spread of cancer. Thus, inflammation offers targets both for the prevention and for cancer treatment. Thus, inflammaging represents the biological phenomena able to couple ageing process with cancer development [22, 23].

### The series

To update some aspects of immune response in elderly, four reviews have been assembled in this series.

Leonardi et al., [18] have reviewed the molecular and cellular pathways involved in age-related chronic inflammation along with their potential triggers and their connection with cancer development. In particular, concerning diet and inflammation, they report that DII significantly correlates with an increased risk of postmenopausal breast cancer, colorectal cancer, lung cancer in smokers, non-Hodgkin lymphoma, bladder cancer, and nasopharyngeal carcinoma. Given the evidence discussed above, it appears plausible to attempt dietary interventions also with food supplements to promote long-term systemic modulation of chronic low-grade inflammation process as an anticancer strategy and towards the improvement of health status in the elderly.

Di Lorenzo et al., [24] review the immunological and non-immunological mechanisms of allergic diseases in the elderly. In the last years the prevalence of allergic diseases in general population is increased because of environmental changes such as better hygiene, Westernized diet, air pollution, climate changes, and other factors that influence host microbiota. Additional factors are responsible for the increase of allergic diseases in elderly as the presence of several comorbidities that should interfere with the development and the type of allergic reactions. However, immunosenescence plays a central role by modifying response to microbiota and triggering inflamm-ageing. In addition, in elderly there is a shift from Th1 responses vs. Th2, hence favouring allergic responses.

The reviews of Weinberger [25] and Aspinall and Lang [26] discuss the prevention of infectious diseases from two different points of view. The Weinberger’s paper regards vaccination as preventive measure against infections. Whilst vaccination is one of the most effective medical interventions ever introduced and has prevented millions of cases of infections worldwide every year, vaccines are often thought to be less effective in providing protection in this older section of society. One major reason for this statement is again the decline seen in effective immunity in this population. The analysis of the possible methods to reverse this decline is the topic of the second one. One approach has been to try to restore immunity within this population to something akin to that seen in younger individuals. Another has been to take a more practical approach believing that a weaker immune system may be provoked into providing a response if the stimulus is considerably strengthened and enhanced.

## Discussion

Because ageing is an ineluctable process, strategies to live longer in a healthy condition have been the main goal of recent research. So, healthy ageing is the main challenge of the twenty-first century both in Western and developing countries [27].

In October 2013, a group of experts in biology and genetics of ageing developed a consensus related to the discovery and development of safe interventions to slow aging and increase healthy lifespan in humans [27]. As stated in the report of the workshop, accumulating scientific evidence from studies conducted in various models suggests that targeting ageing will not just postpone chronic diseases but also prevent multiple age-associated alterations while extending healthy lifespan. Interventions with the potential to target these pathways safely and to induce protective and rejuvenating responses that increase human health span are becoming available.

As suggested by studies conducted in model organisms, calorie restriction (CR), a reduction in food intake from 20% to 40%, without malnutrition, extends maximum lifespan of various model organisms, including yeast, flies, worms and mammals [28].

Some studies in humans demonstrated that this effect seems to be linked to the quality of food rather than to quantity, so a dietary restriction (DR) rather than a CR, in particular to specific aminoacids, seems to be more effective. The result of these DRs is the down regulation of the so-called nutrient-sensing pathways: the insulin/insulin growth factor(IGF)-1 and mTOR, and the reduction of IGF-1 level, differently from CR in which the level of IGF-1 is unchanged [29].

More interestingly, it was demonstrated that a periodic fasting mimicking diet (FMD) in mice (a very low calorie diet followed for five days) decreases inflammation and cancer incidence, other than neurodegeneration, extending lifespan in a good healthy status [30].

Similar experiment conducted in humans, although preliminary, demonstrated the reduction of some proposed markers of biological aging: CRP, systolic/diastolic blood pressure, and serum lipids. In addition, also IGF-1 and body mass index were reduced in these subjects. Thus, a sort of “rejuvenation” of human immune system and down regulation of ageing processes, after FMD, could be hypothesized, as demonstrated in mice who underwent to prolonged fasting (abstinence from foods but not water for two or more days) upon refeeding [31, 32].

A long life in a healthy, vigorous, youthful body has always been one of humanity’s greatest dreams. In the Editorial and in the related papers, anti-ageing strategies have been discussed based on the control of immune-inflammatory responses, aimed not only to slow the ageing process, but also to delay or avoid the onset of age-related diseases such as cancer or combat infectious diseases. Intriguing there is a connection between these response and nutrient sensing pathways already known to delay age-related diseases and promote longevity [33].

## Abbreviations

CR: Calorie restriction
CRP: C reactive protein
DII: Dietary inflammatory index
DR: Dietary restriction
FMD: Fasting mimicking diet
IGF-1: Insulin growth factor-1
IL-6: Interleukin-6
mTor: Mammalian target of rapamycin
